# Identifying Key Variances in Clinical Pathways Associated With Prolonged Hospital Stays Using Machine Learning and ePath Real-World Data: Model Development and Validation Study

**DOI:** 10.2196/71617

**Published:** 2025-12-01

**Authors:** Saori Tou, Koutarou Matsumoto, Asato Hashinokuchi, Fumihiko Kinoshita, Yasunobu Nohara, Takanori Yamashita, Yoshifumi Wakata, Tomoyoshi Takenaka, Hidehisa Soejima, Tomoharu Yoshizumi, Naoki Nakashima, Masahiro Kamouchi

**Affiliations:** 1 Department of Health Care Administration and Management, Graduate School of Medical Sciences, Kyushu University Fukuoka Japan; 2 Department of Surgery and Science, Graduate School of Medical Sciences, Kyushu University Fukuoka Japan; 3 Big Data Science and Technology, Faculty of Advanced Science and Technology, Kumamoto University Kumamoto Japan; 4 Medical Information Center, Kyushu University Hospital Fukuoka Japan; 5 Department of Medical Informatics, Tokushima University Hospital Tokushima Japan; 6 Institute for Medical Information Research and Analysis, Saiseikai Kumamoto Hospital Kumamoto Japan; 7 Department of Medical Informatics, Graduate School of Medical Sciences, Kyushu University Fukuoka Japan; 8 Center for Cohort Studies, Graduate School of Medical Sciences, Kyushu University Fukuoka Japan

**Keywords:** clinical pathway, hospital stay, machine learning, video-assisted thoracoscopic surgery, artificial intelligence, AI

## Abstract

**Background:**

Prolonged hospital stays can lead to inefficiencies in health care delivery and unnecessary consumption of medical resources.

**Objective:**

This study aimed to identify key clinical variances associated with prolonged length of stay (PLOS) in clinical pathways using a machine learning model trained on real-world data from the ePath system.

**Methods:**

We analyzed data from 480 patients with lung cancer (age: mean 68.3, SD 11.2 years; n=263, 54.8% men) who underwent video-assisted thoracoscopic surgery at a university hospital between 2019 and 2023. PLOS was defined as a hospital stay exceeding 9 days after video-assisted thoracoscopic surgery. The variables collected between admission and 4 days after surgery were examined, and those that showed a significant association with PLOS in univariate analyses (*P*<.01) were selected as predictors. Predictive models were developed using sparse linear regression methods (Lasso, ridge, and elastic net) and decision tree ensembles (random forest and extreme gradient boosting). The data were divided into derivation (earlier study period) and testing (later period) cohorts for temporal validation. The model performance was assessed using the area under the receiver operating characteristic curve, Brier score, and calibration plots. Counterfactual analysis was used to identify key clinical factors influencing PLOS.

**Results:**

A 3D heatmap illustrated the temporal relationships between clinical factors and PLOS based on patient demographics, comorbidities, functional status, surgical details, care processes, medications, and variances recorded from admission to 4 days after surgery. Among the 5 algorithms evaluated, the ridge regression model demonstrated the best performance in terms of both discrimination and calibration. Specifically, it achieved area under the receiver operating characteristic curve values of 0.84 and 0.82 and Brier scores of 0.16 and 0.17 in the derivation and test cohorts, respectively. In the final model, a range of variables, including blood tests, care, patient background, procedures, and clinical variances, were associated with PLOS. Among these, particular emphasis was placed on clinical variances. Counterfactual analysis using the ridge regression model identified 6 key variables strongly linked to PLOS. In order of impact, these were abnormal respiratory sounds, postoperative fever, arrhythmia, impaired ambulation, complications after drain removal, and pulmonary air leaks.

**Conclusions:**

A machine learning–based model using ePath data effectively identified critical variances in the clinical pathways associated with PLOS. This automated tool may enhance clinical decision-making and improve patient management.

## Introduction

Prolonged length of stay (PLOS) remains a persistent challenge in modern health care, contributing to inefficient resource use, increased health care costs, and delayed recovery [1]. To address these concerns, hospitals have adopted strategies such as standardized clinical pathways designed to streamline care and promote timely discharge [2,3]. Despite these efforts, PLOS remains common, particularly among surgical patients, due to factors such as postoperative complications, functional decline, and comorbidities.

Identifying patients at high risk of PLOS is crucial for enabling timely and targeted interventions. However, predicting PLOS in routine clinical settings remains challenging due to the complex and dynamic nature of patient conditions and the multidimensional nature of clinical data. Recent advancements in electronic clinical pathways have enabled systematic, real-time recording of deviations from expected recovery trajectories—referred to as “variances” [2]. These variances may reflect meaningful clinical events and could provide valuable insights for predicting outcomes such as PLOS. Nevertheless, predictive systems that fully leverage variance data remain underdeveloped.

To address this gap, we developed a novel electronic clinical pathway system called “ePath,” which records outcomes, outcome assessments, and associated tasks in a structured format known as the outcomes-assessments-tasks (OAT) unit. ePath integrates seamlessly with electronic medical records via a custom data conversion interface and provides a standardized data model for pathway-based care [4-6].

In this study, we applied machine learning algorithms to real-world ePath data from patients undergoing video-assisted thoracoscopic surgery (VATS) for lung cancer, aiming to develop a robust model to predict PLOS risk. VATS has become widely adopted as a minimally invasive alternative to traditional open thoracotomy and is associated with shorter hospital stays and lower health care costs [7-14]. These cost savings are largely attributed to a reduction in PLOS [11,14]. However, a considerable proportion of patients still experience prolonged hospitalization, highlighting the need for more advanced predictive tools.

The objective of this study was to develop an accurate and clinically actionable PLOS prediction model using machine learning applied to variance data captured by ePath. Establishing such a system could enable timely interventions and support clinical decision-making to reduce PLOS.

## Methods

### Ethical Considerations

The study design was approved by the Certified Review Board of the Clinical Research Network Fukuoka (M23082-00). The requirement for informed consent was waived, as this was a retrospective study using anonymized records. No compensation or incentives were provided to participants because this retrospective study used anonymized data and did not involve direct contact with individuals.

### Study Design

This study included patients with lung cancer hospitalized at Kyushu University Hospital in Fukuoka, Japan, who received treatment via the VATS electronic clinical pathway. The ePath system implemented at Kyushu University Hospital in 2018 has been described in previous publications [4-6]. Inpatient clinical data were electronically collected through ePath and compiled into a dataset for outcome prediction using machine learning models.

### Clinical Outcomes

The clinical outcome of interest, PLOS, was defined as a postoperative hospital stay exceeding 9 days based on the target length of stay outlined in the clinical pathway at Kyushu University Hospital.

### Clinical Variables

Data collected during routine inpatient care were comprehensively recorded in the ePath system and used as variables. ePath is an electronic clinical pathway platform developed to standardize and share clinical pathways across multiple electronic medical record systems [4-6]. It facilitates the systematic collection and analysis of related clinical data, thereby supporting the application of artificial intelligence in routine clinical practice. The variables included baseline patient characteristics, functional status, pharmacotherapy, surgical procedures, care processes, variance, and laboratory results.

Baseline patient characteristics, functional status, care procedures, and pharmacotherapy were obtained from the Diagnosis Procedure Combination database, Japan’s medical billing data system, which comprises forms 1, H, and EF files. Form 1 contains demographic information, diagnoses, and disease severity. The H file provides daily records of physical condition, patient care, and activities of daily living. The EF file details daily pharmacotherapy, surgeries, and other clinical activities.

Care processes, outcomes, and variances were embedded in the electronic clinical pathway and collected using the ePath system. The clinical pathway data comprised OAT and variances for each care process during hospitalization; these were collectively referred to as OAT units [4-6]. The OAT units included expected outcomes, assessments of outcomes, and tasks required for assessments, all of which are interlinked. Deviations from predefined care processes were recorded as variances in the OAT units.

Laboratory results were obtained from the laboratory database using the Standardized Structured Medical Information eXchange, a standardized clinical data export system.

The number of discharges began to increase on postoperative day 5. To minimize the impact of missing values due to discharge and to reduce the risk of reverse causality, we limited the candidate explanatory variables to those available by postoperative day 4. From these, we first excluded variables with high overall missingness or outcome-related missingness, as these could introduce substantial bias. Although regularization methods are designed to mitigate overfitting, our previous research showed that including a large number of variables improved apparent prediction accuracy in the derivation cohort but reduced performance in the validation cohort, likely because of high-dimensional noise. To optimize model performance while maintaining generalizability, we used a 2-step variable selection process that combined the exclusion of variables with high missingness and univariate screening. This hybrid approach enabled us to retain clinically important predictors, reduce noise, and enhance the robustness of the final model [15].

### Study Participants

Between July 2019 and May 2023, a total of 577 patients with lung cancer at Kyushu University Hospital underwent VATS treatment. Daily patient data were collected using the ePath system, resulting in 1562 variables. To reduce potential bias, the selection of both the explanatory variables and patients was based on the method described above. We excluded 18 variables with significant differences in PLOS status and 106 with a missing rate of >10%. Of the remaining variables, from those with a univariate significance level of *P*<.01 between the PLOS groups, 63 variables from the day with the lowest *P* values (from admission to postoperative day 4) were chosen as explanatory variables. Patients with missing data for any of these 63 key variables were excluded, resulting in a final cohort of 480 patients for complete case analysis. Figure S1 in Multimedia Appendix 1 shows the flowchart detailing the variables and patient selection process.

### Machine Learning–Based Model

The PLOS prediction model was developed using machine learning algorithms, including sparse linear regression models (Lasso [16], ridge [17], and elastic net [18]) and decision tree ensemble models (random forest [19] and extreme gradient boosting [XGBoost] [20]), following previous research [15]. These algorithms include L1, L2, and mixed regularization techniques for linear models; random forest as a bagging-based decision tree ensemble model using parallel trees; and XGBoost as a boosting-based decision tree model using sequential trees.

The algorithms used in this study were selected considering their complementary strengths. Real-world clinical datasets often contain variables with multicollinearity. To address this issue, we used regularized regression models (ie, Lasso, ridge, and elastic net), which incorporate penalty terms (based on the absolute values, squares, or both) into their loss functions to suppress variance inflation caused by multicollinearity. We also used tree-based ensemble models (ie, random forest and XGBoost), which are inherently robust to multicollinearity because they do not rely on regression coefficients or the inversion of a design matrix. Additionally, sparse regression models offer interpretable, coefficient-based outputs and perform well with high-dimensional clinical data, whereas tree-based models are capable of capturing nonlinear interactions and assessing variable importance.

For each algorithm, hyperparameters were optimized using grid search with 5-fold cross-validation within the derivation cohort (refer to Multimedia Appendix 2 for details). This process was designed to maximize predictive performance while minimizing the risk of overfitting.

The study period was divided into early and late phases, with April 2022 constituting the boundary due to reimbursement revisions and staff turnover in Japan. To assess the generalizability of the predictive model, we used a temporal validation approach. For temporal validation, patients admitted during the early phase (July 2019 to March 2022) were included in the derivation cohort, whereas those admitted during the late phase (April 2022 to May 2023) were included in the test cohort. The model was developed using data from July 2019 to March 2022 and validated with data from April 2022 to May 2023.

To evaluate model performance, we used the area under the receiver operating characteristic curve (AUROC) to assess discrimination and both calibration plots and Brier scores to evaluate calibration (ie, the agreement between predicted probabilities and observed outcomes). AUROC provides a threshold-independent measure of a model’s ability to distinguish between positive and negative cases. The Brier score quantifies the accuracy of probabilistic predictions, while calibration plots visually assess the alignment between predicted and observed probabilities. These metrics are widely used in clinical prediction research and offer complementary perspectives on model performance.

For internal validation, the derivation data underwent 5-fold cross-validation, and the AUROC was calculated. For external validation, the model developed using the derivation data was applied to the test data to calculate the AUROC. Calibration plots were created by dividing the predicted PLOS probabilities into 10 groups and plotting the average predicted PLOS probability against the observed PLOS rate for each group.

Variable importance was assessed using standardized regression coefficients in sparse regression models, MeanDecreaseAccuracy in a random forest, and Gain in XGBoost. All the variables in each predictive model were ranked by importance, with percentages calculated relative to the highest value. These percentages were then averaged across all machine learning algorithms to determine the top-ranking variables, and the values across the models were compared.

The algorithm and variables with the best predictive performance were selected as the final models. Using this final model, differences in PLOS probabilities were estimated for the test cohort based on the presence or absence of variance within OAT units. Variance variables were selected from the highest-ranking variables in the final model based on their importance. This estimation involved comparing PLOS probabilities, assuming that all patients had variances, with actual variance values.

### Statistical Analysis

Differences in frequency based on PLOS presence were tested using the χ^2^ test or Fisher exact test, while continuous variables were used for the Mann-Whitney *U* test. Differences in the predicted PLOS probabilities (assuming all variances vs actual variances) were evaluated using the Wilcoxon signed-rank test. Machine learning model development and statistical analyses were performed using the R statistical package (version 4.0.5; R Foundation for Statistical Computing). Statistical significance was defined as a 2-sided *P*<.05. The detailed R code is provided in Multimedia Appendix 2.

## Results

### Baseline Characteristics of the Patients

The study included 480 patients with lung cancer, with a mean age of 68.3 (SD 11.2) years, of whom 263 (54.8%) were men. Of the 480 patients, 141 (29.4%) had PLOS (Figure S2 in Multimedia Appendix 1). The baseline characteristics were compared according to the PLOS status (Table 1). The patients with PLOS were older, included a higher proportion of men, and had a higher smoking index. In addition, a lower proportion of patients underwent wedge resection in the PLOS group.

**Table 1:** Baseline characteristics of patients with and without prolonged length of stay (PLOS; n=480).

		Without PLOS (n=339)	With PLOS (n=141)	*P* value	
Age (y), mean (SD)		67.0 (11.4)	71.5 (9.8)	<.001	
Male, n (%)		173 (51)	90 (63.8)	.01	
BMI (kg/m^2^), median (IQR)		23.3 (21.0-25.2)	22.6 (20.9-25.8)	.45	
Diabetes mellitus, n (%)		43 (12.7)	22 (15.6)	.48	
Smoking index, median (IQR)		0 (0-600)	400 (0-900)	<.001	
**Type of surgery, n (%)**					.005
	Lobectomy	134 (39.5)	77 (54.6)		
	Segmentectomy	45 (13.3)	19 (13.5)		
	Wedge resection	160 (47.2)	45 (31.9)		

### Time Course Changes in Variables Related to PLOS

A heatmap was created to visualize the relationship between daily clinical variables and PLOS, highlighting specific variables associated with an increased risk of PLOS. The heatmap provided temporal information on the timing since admission, measurement frequency of each variable, and their association with PLOS. Various factors, including the occurrence of variances (Figure S3 in Multimedia Appendix 1), activities of daily living (Figure S4 in Multimedia Appendix 1), treatments (Figure S5 in Multimedia Appendix 1), medications (Figure S6 in Multimedia Appendix 1), and laboratory results (Figure S7 in Multimedia Appendix 1), were significantly associated with PLOS at different time points.

### Machine Learning–Based Prediction Models for PLOS

**Figure 1:**
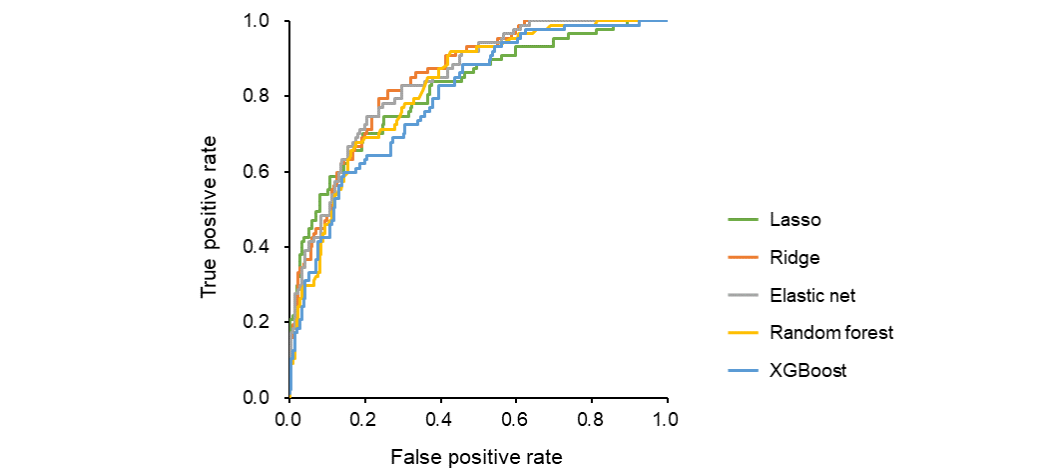
Discriminative performance of the prolonged length of stay (PLOS) prediction model in the derivation cohort. This figure demonstrates the predictive performance of the PLOS prediction model for the derivation cohort of patients admitted in the earlier phase. Variables were selected as predictors from each day between 2 days before VATS and 4 days after surgery based on the lowest *P* value in univariate analysis for each day by PLOS status. The model was developed with 5 machine learning algorithms: Lasso (green), ridge (orange), elastic net (gray), random forest (yellow), and extreme gradient boosting (XGBoost; blue). A 5-fold cross-validation was conducted, and the receiver operating characteristic curve is shown.

**Figure 2:**
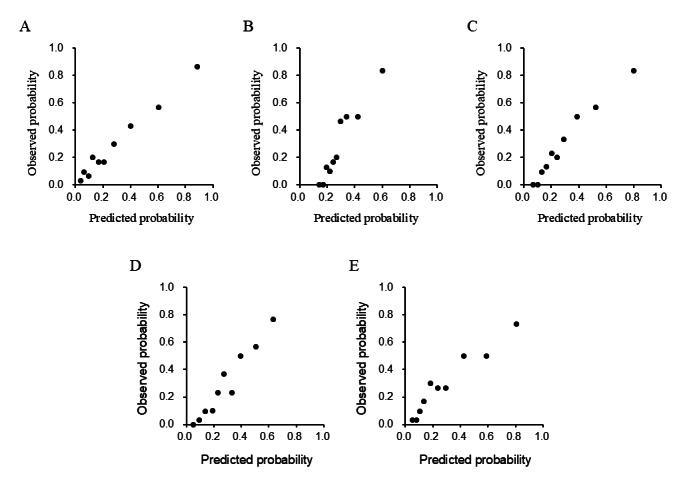
Calibration of the prolonged length of stay (PLOS) prediction model in the derivation cohort. This figure shows the calibration performance of the PLOS prediction model for the derivation cohort of patients admitted in the earlier phase. The model, constructed using 5 machine learning algorithms, namely, Lasso (A), ridge (B), elastic net (C), random forest (D), and extreme gradient boosting (XGBoost; E). The calibration plot divided patients into 10 groups based on predicted PLOS probabilities, with the mean predicted probability on the x-axis and the observed probability on the y-axis.

**Figure 3:**
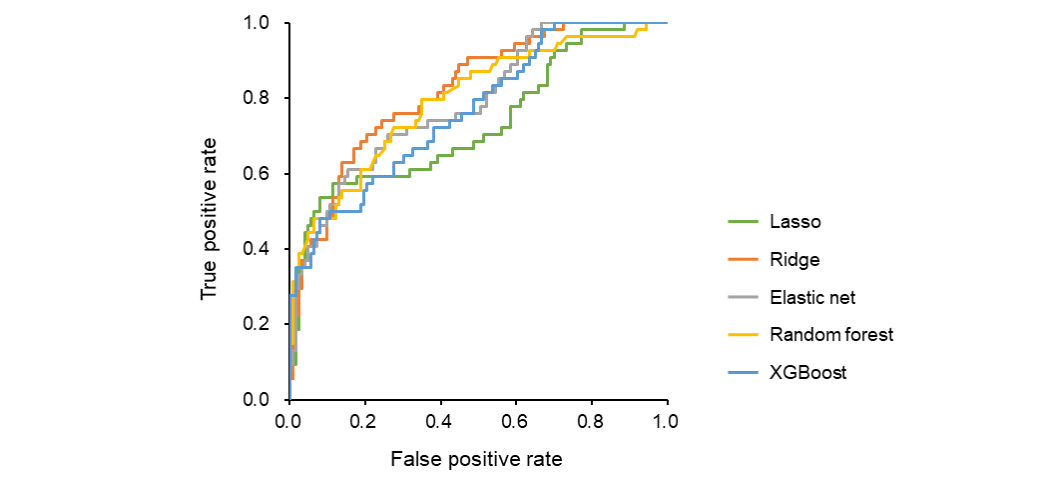
Discriminative performance of the prolonged length of stay (PLOS) prediction model in the test cohort. This figure demonstrates the predictive performance of the PLOS prediction model, initially developed in the derivation cohort, when applied to the temporally validated test cohort of patients admitted in the later phase. The model was developed with 5 machine learning algorithms: Lasso (green), ridge (orange), elastic net (gray), random forest (yellow), and extreme gradient boosting (XGBoost; blue).

**Figure 4:**
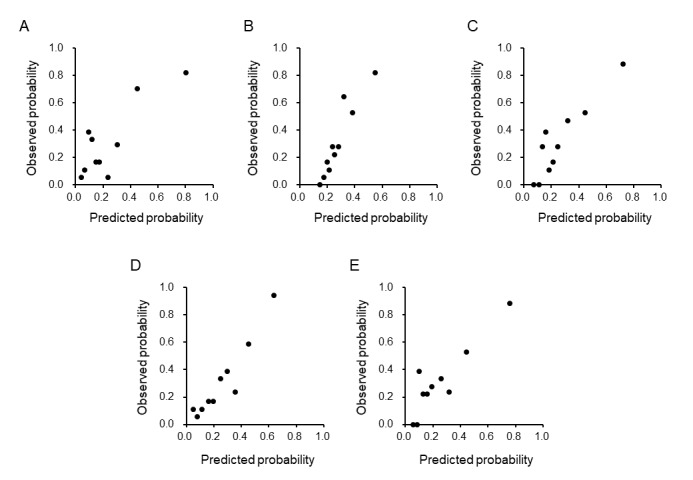
Calibration of the prolonged length of stay (PLOS) prediction model in the test cohort. This figure shows the calibration performance of the PLOS prediction model, initially developed in the derivation cohort, when applied to the temporally validated test cohort of patients admitted in the later phase. The model constructed using 5 machine learning algorithms, namely, Lasso (A), ridge (B), elastic net (C), random forest (D), and extreme gradient boosting (XGBoost; E). The calibration plot divided patients into 10 groups based on predicted PLOS probabilities, with the mean predicted probability on the x-axis and the observed probability on the y-axis.

Five machine learning algorithms were used to develop prediction models in the derivation cohort. The ridge, elastic net, and random forest models showed high discrimination (Figure 1; Multimedia Appendix 3), with calibration plots indicating a good fit (Figure 2) and low Brier scores (Multimedia Appendix 3). These models were validated in the test cohort. Among the models, ridge regression demonstrated the best performance with high discrimination (Figure 3; Multimedia Appendix 4), good calibration (Figure 4), and a low Brier score (Multimedia Appendix 4). Consequently, it was selected as the final model, and the importance of the individual variables was assessed.

### Variable Importance in Machine Learning–Based Prediction Models

We comprehensively analyzed the variable importance identified by the final ridge regression model (Figures S8 and S9 in Multimedia Appendix 1). The ridge regression analysis highlighted several key predictors, such as drain management, respiratory care, use of fibrin sealants, wedge resection, smoking index, age, and levels of C-reactive protein and albumin. Multimedia Appendix 5 shows the importance of the predictors identified by the final model.

### Key Variances Influencing PLOS

**Figure 5:**
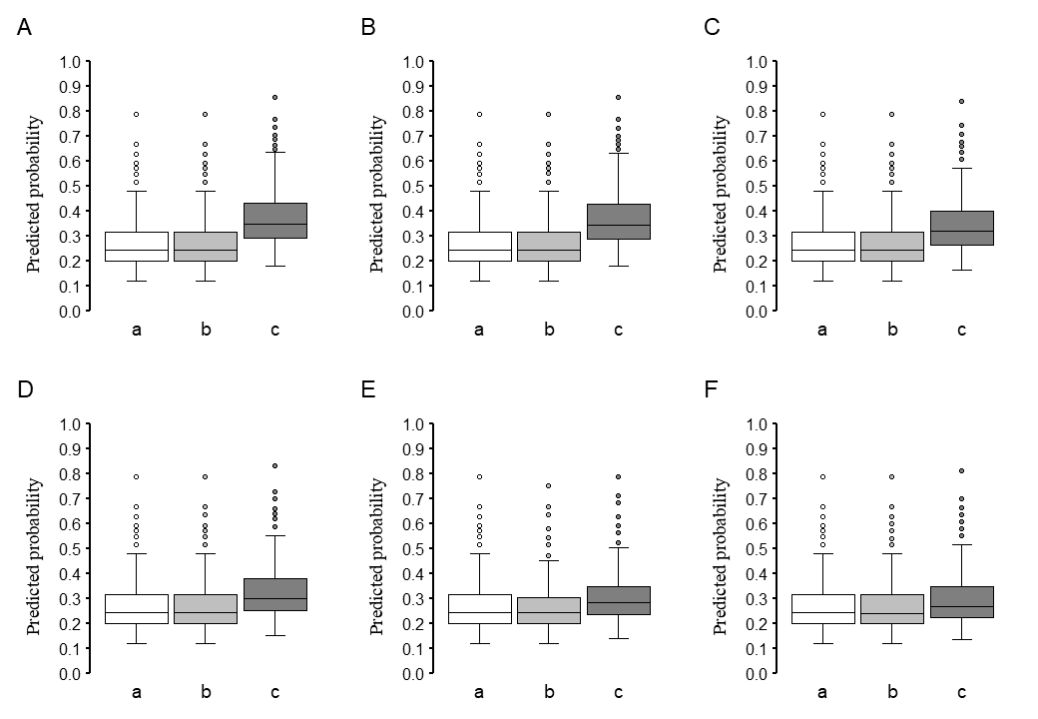
Variance presence and changes in prolonged length of stay (PLOS) probability. This figure shows predicted PLOS probability distributions under different variance conditions: (a) assuming all patients exhibit specific variances ([A] respiratory status: no abnormal breathing sounds; [B] infection: body temperature <37.5 ℃; [C] circulatory status: no arrhythmia; [D] activities of daily living (ADL): able to walk in the ward; [E] drain: no redness, swelling, bleeding, and exudate after drain removal; and [F] drain: no air leak), (b) under actual variance values, and (c) assuming each patient has variances. Box plots show PLOS probabilities for each condition per patient.

**Figure 6:**
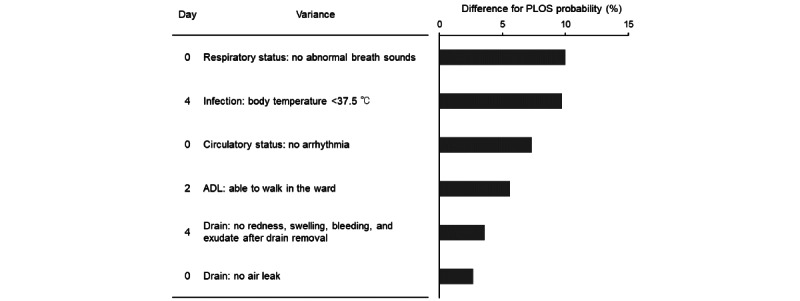
Change in prolonged length of stay (PLOS) probability based on the presence or absence of variances. This figure shows the percentage difference in predicted PLOS probabilities when the PLOS prediction model, developed in the derivation cohort, was applied to the temporally validated test cohort under 2 scenarios: assuming all patients had variances or using the actual presence or absence of variances. The final model was constructed using ridge regression, and the figure illustrates the estimated change in PLOS probability for each variance, assuming all patients exhibit them compared to actual values.

The frequency of clinical variance differed between patients with and without PLOS (Multimedia Appendix 6). Using the final ridge model, we calculated the predicted probability of PLOS for each patient by comparing scenarios assuming a specific variance to be present in all patients with its actual occurrence in individual patients (Figure 5). This analysis identified 6 key postoperative variables whose presence significantly increased the predicted probability of PLOS: abnormal respiratory sounds (*P*<.001), fever (*P*<.001), arrhythmia (*P*<.001), inability to ambulate (*P*<.001), abnormal drain characteristics (*P*<.001), and air leak (*P*=.005; Figure 6). These were identified as critical variances associated with a higher risk of PLOS in this patient population.

## Discussion

### Principal Findings

In this study, we successfully collected comprehensive real-world data using the ePath system from routine clinical practice. A heatmap was used to visualize the timing and emergence of factors potentially associated with PLOS. The importance of the variables, except the top-ranked ones, varied across machine learning algorithms. Temporal validation in the test cohort demonstrated that the ridge regression model performed well in both cohorts. Using this data-driven ridge model, we identified 6 key variances in the clinical pathway that significantly influenced the PLOS risk. Applying machine learning to real-world data allowed us to identify the variances contributing to PLOS, providing a foundation for potential mitigation strategies. This system is promising as a data-driven support tool that leverages electronic information to improve patient care in clinical practice.

### Factors Related to PLOS

Compared with open surgery, VATS is associated with fewer postoperative complications and thus preferred for early-stage lung cancer [7-10,12,13,21]. However, postoperative complications can still arise, leading to potential PLOS [22-24]. Previous studies have identified various factors contributing to PLOS in patients undergoing VATS [25-37]. Many of these factors, such as age [25,27,31,32,34,36,37], male sex [25], BMI [25,31], functional status [25,31], smoking history [25,36], pulmonary function [25,28,31-34], comorbidities [25,26,30-32], tumor status [29,36], and the American Society of Anesthesiologists score [25,32,36], are challenging to modify after admission. Additionally, surgical factors [27-29,31,37], postoperative complications [28,29,37], and chest tube duration [29,30,32] are contributing factors that require further optimization from a surgical perspective.

In real-world settings, addressing modifiable factors before and after VATS is critical for effectively predicting and reducing PLOS. In this study, a heatmap was used to visualize the timing of the relevant factors and their associations with PLOS in the patient cohort. We assessed variable importance in machine learning–based models to identify the key contributors to PLOS risk. The identified factors are consistent with previously reported ones, such as age, smoking history, and surgical techniques (lobectomy and wedge resection), as well as laboratory results indicative of inflammation and malnutrition. Furthermore, we identified additional daily fluctuating factors, described as variances in clinical pathways, that may be associated with postoperative complications. These factors underscore the importance of close monitoring and targeted intervention to enable early and accurate prediction of PLOS and mitigate its impact.

### Key Variances Influencing PLOS

Previous studies on PLOS-related factors have primarily focused on patient characteristics that are not easily modifiable during hospitalization, leading to a lack of clarity regarding specific in-hospital conditions associated with an increased PLOS risk. Moreover, many factors are interrelated, highlighting the need for a comprehensive assessment of how in-hospital conditions affect PLOS risk while adjusting for baseline patient characteristics. Clinical pathways capture variances or deviations from the expected course, enabling the identification of atypical patient trajectories. Recognizing the variances linked to higher PLOS risk can help identify at-risk patients early, optimizing patient care.

Although numerous factors influencing PLOS have been reported in previous studies, their impacts may vary across populations [25-37]. In our cohort, several variables significantly altered the PLOS risk, including postoperative abnormal respiratory sounds, arrhythmia, air leaks, fever, reduced mobility, and abnormal drain characteristics. These variances reflect underlying issues such as pulmonary air leaks, arrhythmias (eg, atrial fibrillation), postoperative infections, and functional impairments.

The emergence of variances may contribute to PLOS through these underlying causes. For instance, a prolonged air leak may delay drain removal, arrhythmias and related complications may necessitate prolonged treatment, infections may require extended use of antibiotics and additional testing, and decreased activities of daily living may slow rehabilitation progress. Although these variances may also be interrelated, identifying and addressing the underlying causes of the 6 key variances may help reduce the risk of PLOS.

Once a variance occurs, prompt clinical intervention is essential. Evaluation of potential causes may include chest x-rays, blood tests (eg, arterial blood gas analysis, inflammatory markers, and electrolyte panels), and inspection of the drainage system. Interventions may include oxygen therapy, postural adjustments, enforced rest, infection control (eg, wound irrigation, disinfection, and dressing changes), administration of antiarrhythmic medications, early mobilization, antibiotic therapy for infections, or pleurodesis for persistent air leaks. Implementing a structured, variance-specific response protocol that integrates timely assessment and targeted intervention may be critical in minimizing PLOS.

Nonmodifiable factors during hospitalization, such as age and comorbidities, are beyond the scope of immediate interventions. However, by understanding how deviations from typical recovery (ie, variance) impact PLOS risk, we can target these specific variances for early intervention. By quantifying changes in the predicted PLOS probability under hypothetical scenarios of variance, we can objectively assess their impact on patient outcomes. Counterfactual analyses simulating changes in outcome probabilities may help estimate population-level effects, paving the way for clinical improvements using the ePath system.

When key variances associated with PLOS are identified, feedback should be provided to the medical team, followed by discussions and consideration of potential improvements. Wherever possible, the underlying causes of each variance should be addressed to prevent recurrence. For variances with significant clinical impact or those amenable to effective intervention, specific management strategies should be developed. Strengthening interprofessional collaboration, providing staff education on early response protocols, and evaluating outcomes can all contribute to continuous improvement. In this context, ePath can facilitate a sustainable plan-do-check-act or plan-do-study-act cycle.

In real-world clinical practice, the variances identified through our approach may help reduce PLOS if managed with appropriate clinical responses. For example, a variance response bundle or an actionable protocol triggered by the model could provide a formal structure for these interventions. Assessing whether such structured approaches reduce PLOS is an important next step.

### Clinical Implications

Machine learning and deep learning are being increasingly applied in health care to uncover hidden patterns in complex datasets and generate predictive insights [38-40]. One of their most promising clinical applications is the automated prediction and visualization of outcomes such as PLOS, which can support clinical decision-making and enhance operational efficiency. However, predicting outcomes using real-world clinical data remains challenging due to its unstructured and heterogeneous nature [41].

To address these challenges, we developed the ePath system—an electronic clinical pathway system designed to systematically collect patient-level and care process data through an integrated workflow. In this study, we adopted a data-driven approach to explore potential causes of PLOS. As a result, we were able to confirm several clinical risk factors. Moreover, we provide novel insights by visualizing when and how specific changes in patient status contribute to the risk of PLOS. This was achieved by integrating a prediction model into the ePath system, enabling real-time, patient-specific visualization of PLOS risk. The structured nature of ePath facilitates robust, data-driven analysis and model development. Recently, tools using clustering techniques on real-world clinical data have been developed to support the design of clinical pathways for patients with lung cancer [42]. The integration of real-world data with pathway analysis via electronic health information systems may mark a new era in data-driven health care.

The novelty of our study lies in leveraging the ePath to capture real-time postoperative clinical variances and applying machine learning techniques to identify key factors associated with PLOS. By embedding the predictive models into a dashboard system, clinicians can visualize each patient’s risk and corresponding clinical variances. When high-risk patterns are detected, the system can generate alerts that prompt timely interventions. Early and appropriate responses may help reduce unnecessary hospitalization and improve patient outcomes. In this way, the system has the potential to serve as an effective clinical decision support tool.

Beyond its clinical utility, ePath also offers managerial value. Hospitals can monitor the occurrence of variances and predicted PLOS risks across patient populations in real time. This enables data-driven decision-making for care delivery and supports the implementation of quality improvement initiatives. By tracking length of stay metrics before and after protocol modifications, institutions can assess the effectiveness of interventions and refine care pathways accordingly. Once validated, such interventions can be standardized and scaled across the organization.

It is important to note that factors contributing to PLOS can vary by institution and patient population [25,31] and may include not only clinical indicators but also social and operational factors [34]. Therefore, models developed using an institution’s data—as enabled by ePath—may be particularly valuable for generating context-specific insights and designing tailored interventions.

Nonetheless, several barriers must be addressed to implement such a system effectively. First, a structured data collection infrastructure, such as ePath, must be established and integrated into clinical workflows. Second, model transparency is essential for clinicians’ trust. While many machine learning models are often perceived as “black boxes,” we selected ridge regression (L2 regularization) as our final model due to its strong performance and interpretability. This model effectively handles multicollinearity and provides coefficients with clear direction and magnitude, which are easily interpretable in clinical contexts.

To further enhance interpretability, we used visualization techniques such as standardized coefficients and variable importance heatmaps. These tools can assist clinicians in understanding model outputs and recognizing the clinical relevance of key variances. Ultimately, the integration of predictive outputs into actionable clinical workflows—through protocols linked to specific alerts—will be essential for translating data insights into meaningful improvements in patient care.

### Limitations

This study has several limitations. Because it used real-world data, issues such as missing values persisted even after dataset preprocessing. In this study, 97 patients were excluded from the complete case analysis (Multimedia Appendix 7), which may have introduced potential selection bias. Additionally, the misclassification of variables cannot be ruled out, which may introduce bias. Given the use of machine learning models, caution is required to address potential overfitting, which may reduce the generalizability of the results. Although we used methods such as 5-fold cross-validation to enhance generalizability and conducted temporal validation, the developed model may not be fully applicable to other patient populations. Moreover, to minimize overfitting from excessive noise, we performed initial variable selection by excluding potentially noncontributory variables. However, this approach may have excluded predictors that were not significant in univariate analyses but could become significant in a multivariate context, potentially reducing predictive accuracy. Further research is warranted to refine variable selection methods that address this methodological trade-off. Differences in health care systems, especially with Japan’s universal health insurance system leading to longer hospital stays than international standards, could also affect the defined outcomes. The postoperative length of stay following VATS varies across countries. Numerous studies have reported a median hospital stay of 4 to 6 days after VATS [25-37]. Although the definition of PLOS differs in the literature—ranging from 2 to 14 days after surgery—it is most commonly set at 4 to 7 days. In this study, the median length of stay was 8 (IQR 7-10) days, and we defined PLOS as 9 days or more based on our institutional clinical pathway target. This threshold may be longer than those used in other countries, and therefore, cross-national comparisons should be interpreted with caution. Hospital length of stay is influenced by a wide range of factors that vary across hospitals, regions, and countries. In addition to patient-level clinical characteristics, institutional and systemic factors can significantly impact length of stay. These include health care financing and reimbursement systems, the availability of regional health care resources, hospital size and function, clinical staff expertise, adherence to clinical guidelines, the robustness of discharge planning infrastructure, access to postacute care services, and the presence of family support systems. This study was based on data from one university hospital, which may not be representative of the general hospital population. To enhance the generalizability of our findings, future research should include model retraining and validation using multicenter datasets, the incorporation of health care system–related variables into the models, and the development of customizable models that can be adapted to specific institutional or regional contexts.

### Conclusions

Using the ePath system, we systematically collected real-world data and developed machine learning models to predict PLOS in a data-driven manner. Successful implementation of data-driven PLOS prediction for specific patient populations could serve as a valuable tool for evidence-based quality improvement in health care. However, additional validation is necessary to implement and operationalize these prediction systems in real-world clinical environments.

## Data Availability

The datasets analyzed during this study are not publicly available due the confidential nature of the data but are available from the corresponding author on reasonable request.

## Appendix

Multimedia Appendix 1 Supplementary figures.

Multimedia Appendix 2 Supplementary information on methods.

Multimedia Appendix 3 Area under the receiver operating characteristic curve and Brier score of prolonged length of stay prediction models in derivation cohorts.

Multimedia Appendix 4 Area under the receiver operating characteristic curve and Brier score of prolonged length of stay prediction models in test cohort.

Multimedia Appendix 5 Variable importance for predicting prolonged length of stay in the ridge regression model.

Multimedia Appendix 6 Frequency of variance by prolonged length of stay.

Multimedia Appendix 7 Baseline characteristics of included and excluded patients.

## Abbreviations

AUROC: area under the receiver operating characteristic curve
XGBoost: extreme gradient boosting
OAT: outcomes-assessments-tasks
PLOS: prolonged length of stay
VATS: video-assisted thoracoscopic surgery
