# Supplementary material for: Identifying Key Variances in Clinical Pathways Associated With Prolonged Hospital Stays Using Machine Learning and ePath Real-World Data: Model Development and Validation Study
Source: JMIR Med Inform. 2025 Dec 1;13:e71617. doi: 10.2196/71617 (PMC12706448; doi:10.2196/71617)
Supplement: Multimedia Appendix 1 [file medinform_v13i1e71617_app1.docx]

**Figure S1. Variable selection flowchart**


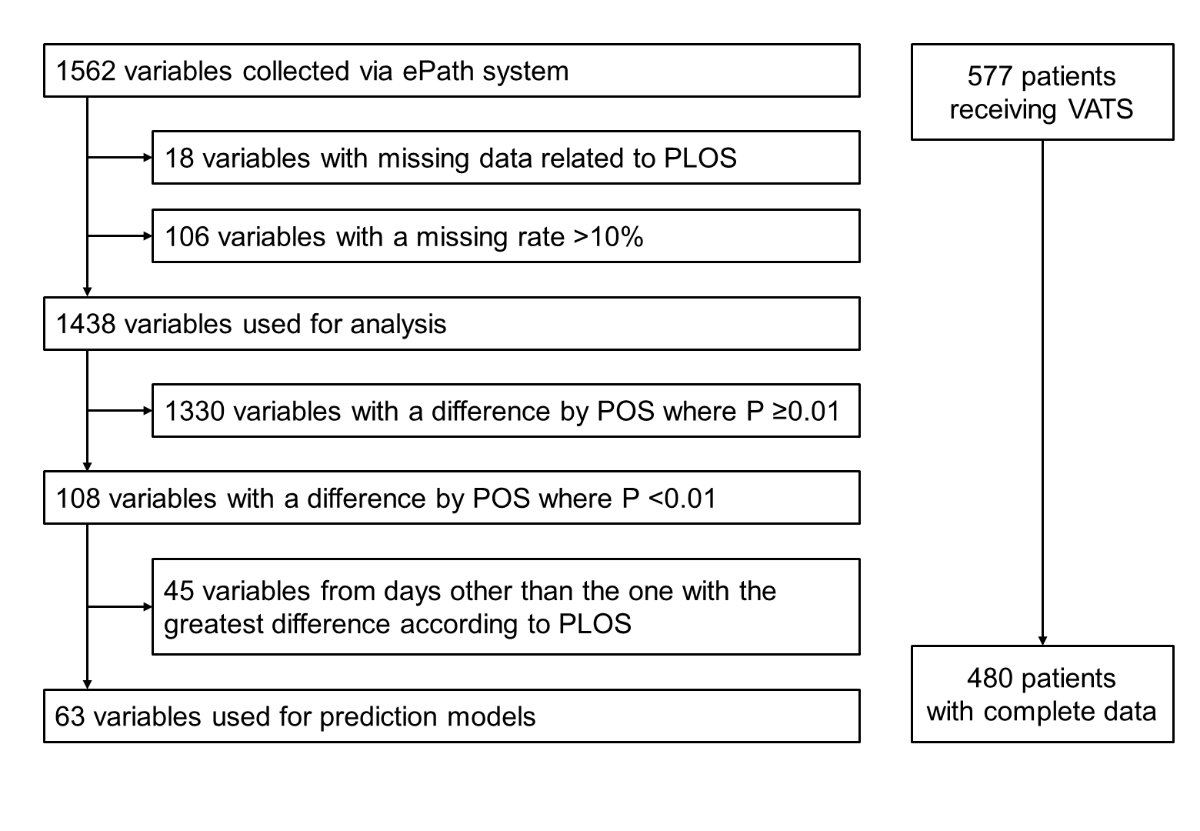


This flowchart details the selection process of the variables and patients included in the analysis. Initially, 18 variables with missing values related to PLOS and 106 variables with a missing rate exceeding 10% were excluded, resulting in 1,438 variables. An additional 1,330 variables with a univariate P-value ≥0.01 for PLOS status were excluded. From the remaining multiple-day variables, those with the most significant difference were selected, yielding 63 variables for the prediction model. Of the 577 patients with lung cancer who underwent VATS, 480 without missing data for these 63 variables were included in the complete case analysis. PLOS: prolonged length of stay; VATS: video-assisted thoracoscopic surgery.

**Figure S2. Distribution of the length of hospital stay**


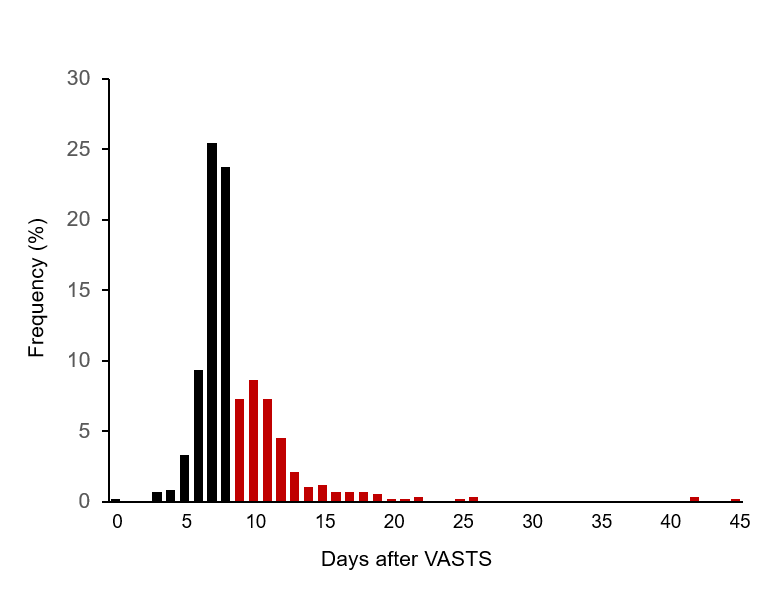


This histogram shows the distribution of discharged patients based on the number of days from VATS to discharge. X-axis shows the number of days post-VATS and y-axis shows the percentage of patients discharged each day. The section representing the PLOS is highlighted in red. PLOS: prolonged length of stay; VATS: video-assisted thoracoscopic surgery.

**Figures S3. Variance occurrence and PLOS**


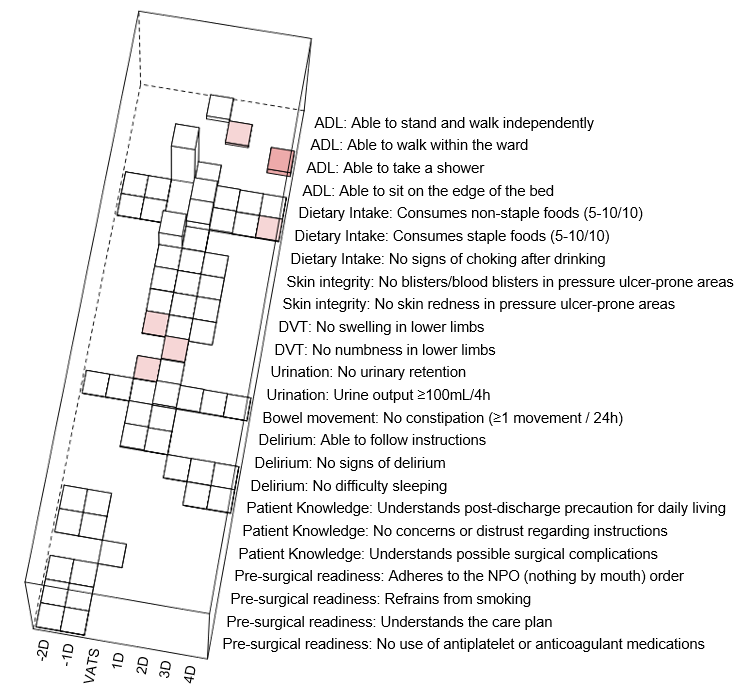

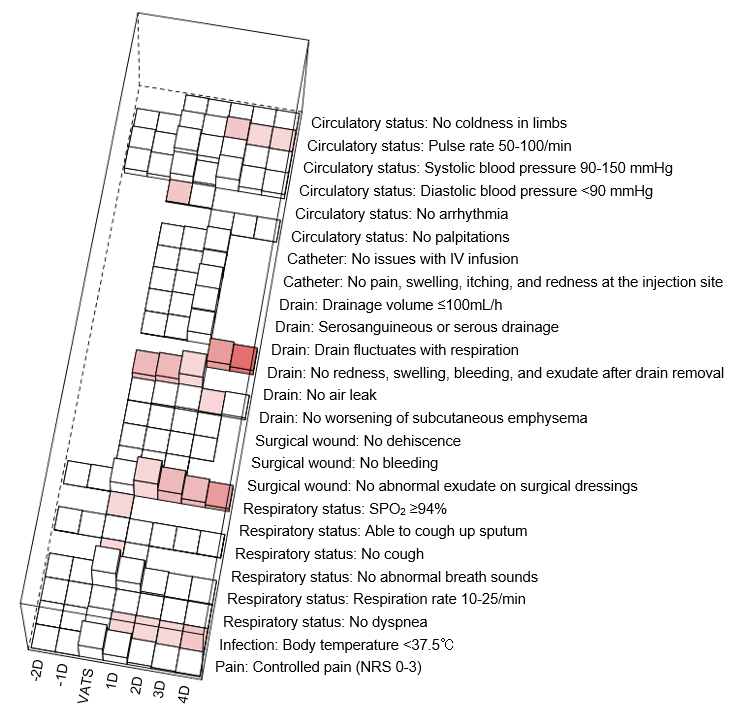


The heat maps show the occurrence of variances from two days before surgery (-2D) to the day of VATS and four days postoperatively (4D), as well as the association between each variance and PLOS. The y-axis represents each variance, x-axis represents each day relative to VATS, and the z-axis represents the frequency of variance occurrence. P-values from the association tests with PLOS are shown in red, with darker shades indicating lower P-values. PLOS: prolonged length of stay; VATS: video-assisted thoracoscopic surgery.

**Figure S4. Activities of daily living and PLOS**


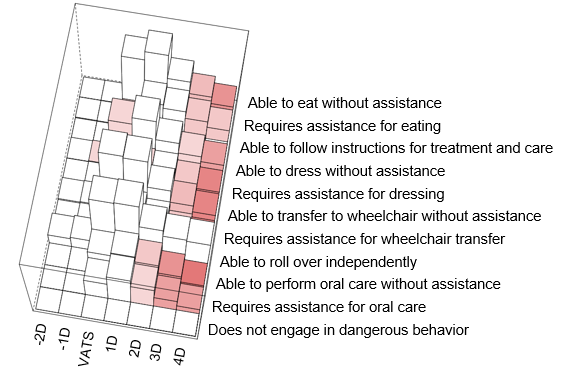


The heatmap shows activities of daily living (ADL) levels from two days before surgery (-2D) to the day of VATS and four days postoperatively (4D), as well as the association between each ADL level and PLOS. The Y-axis represents each ADL level, x-axis represents each day relative to VATS, and the Z-axis represents the average level of impairment for each ADL, with higher values indicating greater impairment. P-values from the association tests with PLOS are shown in red, with darker shades indicating lower P-values. PLOS: prolonged length of stay; VATS: video-assisted thoracoscopic surgery.

**Figure S5. Treatment and PLOS**


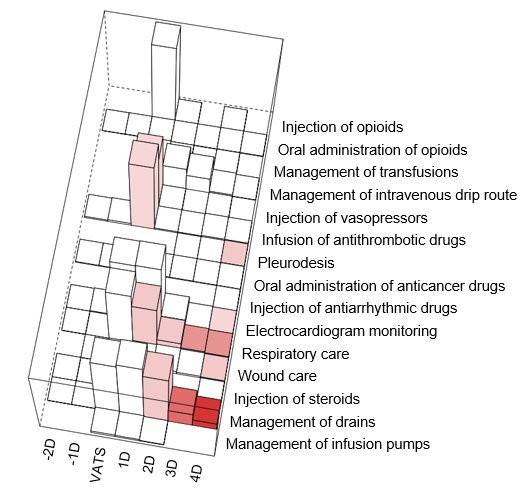


The heatmap shows the implementation of treatments from two days before surgery (-2D) to the day of VATS and four days postoperatively (4D), as well as the association between each treatment and PLOS. The Y-axis represents each treatment, the x-axis represents each day relative to VATS, and the Z-axis represents the frequency of treatment implementation. P-values from the association tests with PLOS are shown in red, with darker shades indicating lower P-values. PLOS: prolonged length of stay; VATS: video-assisted thoracoscopic surgery.

**Figure S6. Medication and PLOS**


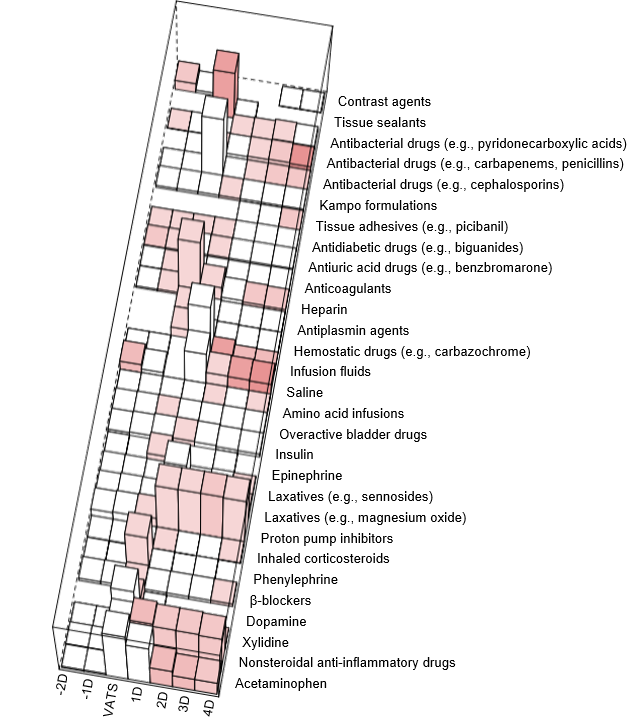


The heatmap shows the implementation of medications from two days before surgery (-2D) to the day of VATS and four days postoperatively (4D), as well as the association between each medication and PLOS. The Y-axis represents each medication, the x-axis represents each day relative to VATS, and the Z-axis represents the frequency of medication administration. P-values from the association tests with PLOS are shown in red, with darker shades indicating lower P-values. PLOS: prolonged length of stay; VATS: video-assisted thoracoscopic surgery.

**Figure S7. Blood test results and PLOS**


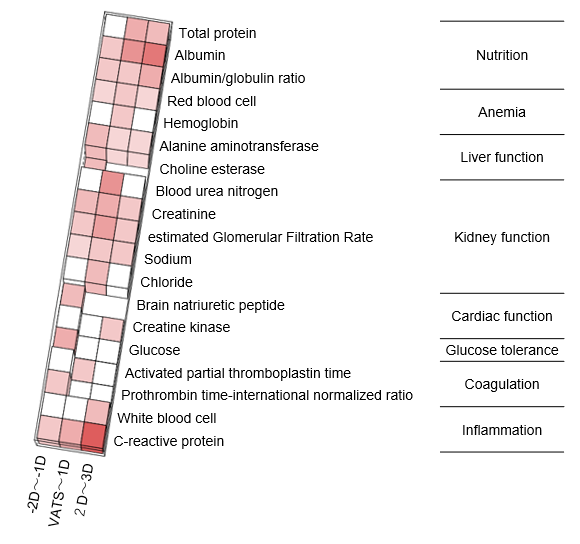


The heatmap shows the implementation of blood tests from two days before surgery (-2D) to the day of VATS and four days postoperatively (4D), as well as the association between each blood test result and PLOS. The Y-axis represents each blood test item, the x-axis represents each day relative to VATS, and the Z-axis represents the frequency of blood test implementation. P-values from the association tests with PLOS are shown in red, with darker shades indicating lower P-values. PLOS: prolonged length of stay; VATS: video-assisted thoracoscopic surgery.

**Figure S8. Variable importance of care in the PLOS prediction model**

This figure displays the importance of care-related variables (EF file data) in the PLOS prediction model developed using ridge regression in the derivation cohort. Variable importance was shown as a percentage, with the most important variable set at 100%. The timeline for variable acquisition spans two days before (-2D) to four days after VATS (4D). PLOS: prolonged length of stay; VATS: video-assisted thoracoscopic surgery.

**Figure S9. Variable importance of baseline data, blood test results, and variance in the PLOS prediction model**

This figure displays the importance of baseline data, blood test results (laboratory data), and variance in the PLOS prediction model developed using ridge regression in the derivation cohort. Variable importance was shown as a percentage, with the most important variable set at 100%. The timeline for variable acquisition spans two days before (-2D) to four days after VATS (4D). PLOS: prolonged length of stay; VATS: video-assisted thoracoscopic surgery.
