# Supplementary material for: Identifying Key Variances in Clinical Pathways Associated With Prolonged Hospital Stays Using Machine Learning and ePath Real-World Data: Model Development and Validation Study
Source: JMIR Med Inform. 2025 Dec 1;13:e71617. doi: 10.2196/71617 (PMC12706448; doi:10.2196/71617)
Supplement: Multimedia Appendix 2 [file medinform_v13i1e71617_app2.docx]

**Methods**

***R programs for model training process***

Data analysis was conducted using RStudio (version 2023.06.0, http://www.rstudio.com/) and the R statistical software package (version 4.0.5, http://www.r-project.org/). The Least Absolute Shrinkage and Selection Operator (LASSO), ridge regression, and Elastic Net were implemented using the glmnet package (https://cran.r-project.org/web/packages/glmnet/glmnet.pdf). Parameter tuning for λ was conducted using grid search combined with five-fold cross-validation, incrementing λ from 0 to 1 in steps of 0.01. For Elastic Net, both α and λ were tuned within the same range and increments. The random forest model was implemented using the random forest package (https://cran.r-project.org/web/packages/randomForest/randomForest.pdf). The hyperparameter mtry, which specifies the number of variables randomly sampled at each tree split, was tuned between 1 and 10 using a grid search combined with five-fold cross-validation. A gradient-boosting decision tree model was developed using the xgboost package (https://cran.r-project.org/web/packages/xgboost/xgboost.pdf). For hyperparameters, max_depth (tree depth), subsample (proportion of training data used for each boosting iteration), and colsample_bytree (proportion of features randomly selected for each tree) were tuned within the ranges of 1–10 and 0.8–1, respectively. Additionally, nrounds was set to a maximum of 5,000, with early_stopping_rounds set to 10, and training was terminated if the validation performance did not improve for 10 consecutive rounds. Other parameters were fixed as follows: gamma = 0, eta = 0.1, and min_child_weight = 1. Variable importance was assessed using different metrics for each model. Standardized regression coefficients were used for ridge regression, LASSO, and elastic nets. The random forest model evaluates variable importance using the mean decrease in accuracy, whereas the gradient-boosting decision-tree model uses the gain metric.

***R programs for prediction metrics and validation***

The “pROC” (https://cran.r-project.org/web/packages/pROC/pROC.pdf) package was utilized to calculate the area under the receiver operating characteristic curve (AUROC). In addition, Brier score was calculated as the average squared difference between the predicted probabilities and observed outcomes. For internal validation, the R package “rsample” (https://cran.r-project.org/web/packages/rsample/rsample.pdf) was used with a stratified five-fold cross-validation.
