# Supplementary material for: Identifying Key Variances in Clinical Pathways Associated With Prolonged Hospital Stays Using Machine Learning and ePath Real-World Data: Model Development and Validation Study
Source: JMIR Med Inform. 2025 Dec 1;13:e71617. doi: 10.2196/71617 (PMC12706448; doi:10.2196/71617)
Supplement: Multimedia Appendix 3 [file medinform_v13i1e71617_app3.docx]

**Table S1. AUROC and Brier score of PLOS prediction models in derivation cohorts**

|  | AUROC, mean±SD | Brier score |
| --- | --- | --- |
| Lasso | 0.819±0.057 | 0.142 |
| Ridge | 0.840±0.070 | 0.155 |
| Elastic Net | 0.839±0.061 | 0.142 |
| Random forest | 0.825±0.058 | 0.153 |
| XGBoost | 0.803±0.053 | 0.158 |

A prediction model was constructed using the early study period cohort as the training and validation cohorts. PLOS was defined as hospital stay exceeding seven days post-surgery. Model performance was evaluated using the AUROC for discrimination and the Brier score for calibration. The early cohort of the study period was divided into five folds for cross-validation, the model built on the training data was evaluated using the validation data, and the results were averaged. PLOS: prolonged length of hospital stay; AUROC: area under the receiver operating characteristic curve.
