# Supplementary material for: Identifying Key Variances in Clinical Pathways Associated With Prolonged Hospital Stays Using Machine Learning and ePath Real-World Data: Model Development and Validation Study
Source: JMIR Med Inform. 2025 Dec 1;13:e71617. doi: 10.2196/71617 (PMC12706448; doi:10.2196/71617)
Supplement: Multimedia Appendix 4 [file medinform_v13i1e71617_app4.docx]

**Table S2. AUROC and Brier score of PLOS prediction models in test cohort**

|  | AUROC | Brier score |
| --- | --- | --- |
| Lasso | 0.730 | 0.171 |
| Ridge | 0.820 | 0.171 |
| Elastic Net | 0.788 | 0.163 |
| Random forest | 0.794 | 0.340 |
| XGBoost | 0.766 | 0.437 |

A prediction model was constructed using the early cohort of the study period and temporal validation was performed using a later test cohort. PLOS was defined as hospital stay exceeding nine days post-surgery. Model performance was evaluated using the AUROC for discrimination and the Brier score for calibration. PLOS: prolonged length of stay; AUROC: area under the receiver operating characteristic curve.
