# Supplementary material for: Identifying Key Variances in Clinical Pathways Associated With Prolonged Hospital Stays Using Machine Learning and ePath Real-World Data: Model Development and Validation Study
Source: JMIR Med Inform. 2025 Dec 1;13:e71617. doi: 10.2196/71617 (PMC12706448; doi:10.2196/71617)
Supplement: Multimedia Appendix 5 [file medinform_v13i1e71617_app5.docx]

**Table S3. Variable importance for predicting PLOS in the ridge regression model**

| Data | Factor | % |
| --- | --- | --- |
| C (4) | Management of drain | 100.0 |
| C (3) | Respiratory care | 60.6 |
| C (0) | Fibrin sealants | 56.9 |
| B (0–3) | C-reactive protein | 50.4 |
| B (0–3) | Albumin | 49.0 |
| S (–) | Wedge resection | 48.9 |
| P (–) | Smoking index | 44.7 |
| C (2) | Acetaminophen | 44.5 |
| V (4) | Drain: No redness, swelling, bleeding, and exudate after drain removal | 44.1 |
| C (2) | Antibacterial drugs (e.g., pyridonecarboxylic acid) | 43.9 |
| C (2) | Contrast agents | 43.5 |
| C (2) | Infusion fluids | 41.3 |
| C (4) | Antibacterial drugs (e.g., cephems) | 40.3 |
| V (2) | ADL: Able to walk in the ward | 40.0 |
| B (0–3) | Total protein | 40.0 |
| V (0) | Drain: No air leak | 38.9 |
| V (4) | Infection: Body temperature <37.5℃ | 38.9 |
| V (0) | Circulatory Status: No arrhythmia | 38.3 |
| C (4) | Laxatives (e.g., sennoside) | 37.6 |
| S (–) | Lobectomy | 35.7 |
| B (0–3) | Albumin/globulin ratio | 34.6 |
| C (0) | Dopamine | 34.3 |
| C (0) | Wound care | 34.2 |
| S (–) | Sequel of cerebrovascular disease | 33.9 |
| V (0) | Respiratory Status: No abnormal breath sounds | 32.6 |
| C (4) | Amino acid infusion | 32.3 |
| C (3) | Laxatives (e.g., magnesium oxide) | 29.9 |
| B (0–1) | Creatinine | 28.3 |
| P (–) | Age | 25.5 |
| C (0) | Able to follow instructions for treatment and care | 25.5 |
| B (0–1) | Red blood cell | 21.3 |

This demonstrates the importance of the variables used in ridge regression for the PLOS prediction model constructed for the derivation cohort. Among the variables occurring from two days before surgery (-2D) to four days after surgery (4D), standardized regression coefficients for PLOS risk were calculated. Then, the variable importance of each predictor was expressed as a percentage, calculated by taking the standardized regression coefficient of each variable relative to the largest standardized regression coefficient among all variables, which is set at 100%. The “Data” column specifies the data source from which the variable was obtained (B: blood test, C: care, P: patient background, S: surgery, V: variance) and the day on which the variable was obtained from two days before (-2D) to four days (4D) after the video-assisted thoracoscopic surgery (VATS).
