# Supplementary material for: Identifying Key Variances in Clinical Pathways Associated With Prolonged Hospital Stays Using Machine Learning and ePath Real-World Data: Model Development and Validation Study
Source: JMIR Med Inform. 2025 Dec 1;13:e71617. doi: 10.2196/71617 (PMC12706448; doi:10.2196/71617)
Supplement: Multimedia Appendix 6 [file medinform_v13i1e71617_app6.docx]

**Table S4. Frequency of variance by PLOS**

| Variance | No PLOS | PLOS | P |
| --- | --- | --- | --- |
| ADL |  |  |  |
| Able to stand up and walk | 12/339 (3.5) | 9/141 (6.4) | 0.25 |
| Able to walk in the ward | 5/339 (1.5) | 7/141 (5.0) | 0.047 |
| Able to take a shower | 10/339 (2.9) | 18/139 (12.9) | <0.001 |
| Able to sit on the edge of the bed | 154/314 (49) | 69/132 (52.3) | 0.60 |
| Dietary intake |  |  |  |
| Consumes non-staple food (5–10/10) | 38/1958 (1.9) | 23/820 (2.8) | 0.20 |
| Consumes staple food (5–10/10) | 39/1958 (2.0) | 25/820 (3.0) | 0.12 |
| No choking after drinking | 64/674 (9.5) | 30/279 (10.8) | 0.64 |
| Decubitus |  |  |  |
| No blisters/blood blisters  on pressure ulcer-prone areas | 1/1017 (0.1) | 2/423 (0.5) | 0.21 |
| No skin redness on pressure ulcer-prone areas | 2/1017 (0.2) | 3/423 (0.7) | 0.15 |
| DVT |  |  |  |
| No swelling in lower limbs | 1/1017 (0.1) | 2/423 (0.5) | 0.21 |
| No numbness in lower limbs | 0/1017 (0.0) | 3/423 (0.7) | 0.03 |
| Urination |  |  |  |
| No urinary retention | 5/339 (1.5) | 7/141 (5.0) | 0.047 |
| Urine volume ≥100mL/4h | 2/678 (0.3) | 7/282 (2.5) | 0.004 |
| Defecation |  |  |  |
| No constipation (≥1/24h) | 18/1938 (0.9) | 12/812 (1.5) | 0.29 |
| Delirium |  |  |  |
| Able to follow instructions | 2/678 (0.3) | 2/282 (0.7) | 0.59 |
| No signs of delirium | 7/678 (1.0) | 3/282 (1.1) | 1.00 |
| No difficulty sleeping | 5/1014 (0.5) | 3/419 (0.7) | 0.70 |
| Patient knowledge |  |  |  |
| Understands precaution for daily living after discharge | 2/678 (0.3) | 4/282 (1.4) | 0.07 |
| No questions or distrust regarding instructions | 0/347 (0.0) | 0/144 (0.0) | – |
| Understands surgical complications | 0/347 (0.0) | 0/144 (0.0) | – |
| Readiness for surgery |  |  |  |
| Adheres to the NPO order | 0/327 (0.0) | 0/135 (0.0) | – |
| Refrains from smoking | 1/441 (0.2) | 0/182 (0.0) | 1.00 |
| Understands the care plan | 1/603 (0.2) | 0/256 (0.0) | 1.00 |
| No use of antiplatelet or anticoagulant medications | 1/603 (0.2) | 2/256 (0.8) | 0.21 |
| Circulatory status |  |  |  |
| No coldness of limbs | 0/1695 (0.0) | 2/705 (0.3) | 0.09 |
| Pulse rate 50–100/min | 113/2299 (4.9) | 90/961 (9.4) | <0.001 |
| Systolic blood pressure 90–150 mmHg | 189/2300 (8.2) | 90/963 (9.3) | 0.33 |
| Diastolic blood pressure <90 mmHg | 208/2089 (10.0) | 69/852 (8.1) | 0.13 |
| No arrhythmia | 3/678 (0.4) | 9/282 (3.2) | 0.001 |
| No palpitations | 0/1017 (0.0) | 0/423 (0.0) | – |
| Catheter |  |  |  |
| No issues with drip infusion | 35/1017 (3.4) | 8/423 (1.9) | 0.16 |
| No pain, swelling, itching, and redness at the injection site | 26/1017 (2.6) | 8/423 (1.9) | 0.57 |
| Drain |  |  |  |
| Drainage volume ≤100mL/h | 71/1014 (7.0) | 24/423 (5.7) | 0.42 |
| Serosanguineous or serous drainage | 75/1014 (7.4) | 26/423 (6.1) | 0.46 |
| Respiratory fluctuation | 66/1014 (6.5) | 23/423 (5.4) | 0.52 |
| No redness, swelling, bleeding, and exudate after drain removal | 33/678 (4.9) | 56/282 (19.9) | <0.001 |
| No air leak | 110/1013 (10.9) | 95/423 (22.5) | <0.001 |
| No worsening of subcutaneous emphysema | 30/1685 (1.8) | 27/697 (3.9) | 0.004 |
| Surgical Wound |  |  |  |
| No dehiscence | 0/1356 (0.0) | 2/564 (0.4) | 0.09 |
| No bleeding | 1/1356 (0.1) | 2/564 (0.4) | 0.21 |
| No abnormal exudate on surgical gauze | 3/1017 (0.3) | 5/423 (1.2) | 0.05 |
| Respiratory Status |  |  |  |
| SPO_2_ ≥94% | 235/2297 (10.2) | 170/961 (17.7) | <0.001 |
| Able to cough up sputum | 1/339 (0.3) | 4/141 (2.8) | 0.03 |
| No cough | 7/2298 (0.3) | 4/961 (0.4) | 0.74 |
| No abnormal breath sounds | 0/678 (0.0) | 3/282 (1.1) | 0.03 |
| Respiration rate 10–25/min | 204/2298 (8.9) | 76/961 (7.9) | 0.41 |
| No dyspnea | 3/2298 (0.1) | 5/961 (0.5) | 0.05 |
| Infection |  |  |  |
| Body temperature <37.5℃ | 32/2297 (1.4) | 33/961 (3.4) | <0.001 |
| Pain |  |  |  |
| Controlled pain (NRS 0–3) | 170/2297 (7.4) | 67/961 (7.0) | 0.72 |

The total number/measurement frequency (%) of variances occurring from two days before surgery to four days after surgery (4D) was compared based on the presence or absence of a prolonged length of stay (PLOS). Frequency was calculated using the number of evaluations as the denominator and the number of occurrences of variance as the numerator.
