# Supplementary material for: Identifying Key Variances in Clinical Pathways Associated With Prolonged Hospital Stays Using Machine Learning and ePath Real-World Data: Model Development and Validation Study
Source: JMIR Med Inform. 2025 Dec 1;13:e71617. doi: 10.2196/71617 (PMC12706448; doi:10.2196/71617)
Supplement: Multimedia Appendix 7 [file medinform_v13i1e71617_app7.docx]

**Table S5. Baseline characteristics of included and excluded patients.**

|  | Included n=480 | Excluded n=97 | P-value |
| --- | --- | --- | --- |
| Age, mean ± SD | 68.3 ±11.2 | 69.3±11.2 | 0.43 |
| Male, n (%) | 263 (54.8) | 65 (67.0) | 0.04 |
| Body mass index, kg/m2, median (IQR) | 23.2 (21.0-25.3) | 23.9 (21.3-25.5) | 0.29 |
| Diabetes mellitus, n (%) | 65 (13.5) | 15 (15.5) | 0.73 |
| Smoking index, median (IQR) | 0 (0-701) | 110 (0-808) | 0.45 |
| Type of surgery, n (%) |  |  | 0.72 |
| Lobectomy | 205 (42.7) | 43 (44.3) |  |
| Segmentectomy | 64 (13.3) | 10 (10.3) |  |
| Wedge resection | 211 (44.0) | 44 (45.4) |  |
